# Preclinical Interventions in Mouse Models of Frontotemporal Dementia Due to Progranulin Mutations

**DOI:** 10.1007/s13311-023-01348-6

**Published:** 2023-02-13

**Authors:** Shreya N. Kashyap, Nicholas R. Boyle, Erik D. Roberson

**Affiliations:** Center for Neurodegeneration and Experimental Therapeutics, Alzheimer’s Disease Center, Medical Scientist Training Program, Department of Neurology, University of Alabama at Birmingham, Birmingham, AL 35294 USA

**Keywords:** Frontotemporal dementia, Neurodegeneration, Progranulin, Haploinsufficiency, Lysosome, Gene therapy

## Abstract

Heterozygous loss-of-function mutations in progranulin (*GRN*) cause frontotemporal dementia (FTD), a leading cause of early-onset dementia characterized clinically by behavioral, social, and language deficits. There are currently no FDA-approved therapeutics for FTD-*GRN*, but this has been an active area of investigation, and several approaches are now in clinical trials. Here, we review preclinical development of therapies for FTD-*GRN* with a focus on testing in mouse models. Since most FTD-*GRN-*associated mutations cause progranulin haploinsufficiency, these approaches focus on raising progranulin levels. We begin by considering the disorders associated with altered progranulin levels, and then review the basics of progranulin biology including its lysosomal, neurotrophic, and immunomodulatory functions. We discuss mouse models of progranulin insufficiency and how they have been used in preclinical studies on a variety of therapeutic approaches. These include approaches to raise progranulin expression from the normal allele or facilitate progranulin production by the mutant allele, as well as approaches to directly increase progranulin levels by delivery across the blood–brain barrier or by gene therapy. Several of these approaches have entered clinical trials, providing hope that new therapies for FTD-*GRN* may be the next frontier in the treatment of neurodegenerative disease.

**Supplementary Information:**

The online version contains supplementary material available at 10.1007/s13311-023-01348-6.

The approval of the first amyloid immunotherapies by the US Food and Drug Administration for patients with Alzheimer’s disease, although not without controversy, marks the beginning of a new era in the treatment of neurodegenerative dementias. Therapeutics targeting one of the primary disease-associated molecules are now approved, and a neuropathological hallmark of neurodegenerative dementia can be substantially eliminated from the brains of those affected. Following aducanumab, lecanemab, and other amyloid immunotherapies likely to follow, treatments targeting progranulin for frontotemporal dementia (FTD) may be among the next round of disease-modifying neurodegenerative disease therapeutics.

One advantage in the pursuit of progranulin-targeted therapeutics is that, unlike amyloid-beta, tau, and other proteins associated with neurodegenerative diseases that have gain-of-function effects, the mechanism of progranulin-related disease is clearly loss-of-function. Thus, the approach to progranulin therapeutics can focus on boosting levels of progranulin, which, while not trivial, is likely more straightforward than identifying specific downstream gain-of-function effects along with strategies to selectively block them. Indeed, several progranulin-targeting therapies have already entered clinical trials. Such progress, of course, comes on the heels of rigorous preclinical studies, many of which have utilized mouse models.

Our goal here is to review data from preclinical testing of potential treatments for progranulin-related FTD in mouse models. Before considering studies on specific therapeutic approaches, we begin with a review of the neurologic disorders caused by progranulin insufficiency and other diseases associated with progranulin, key features of progranulin biology, and the currently available mouse models and outcome measures used in preclinical studies on progranulin therapeutics.

## Progranulin-Associated Disorders

### Frontotemporal Dementia

Frontotemporal dementia (FTD), a leading cause of early-onset dementia, is a clinically heterogeneous disorder characterized neuropathologically by protein aggregates [1–7], neuroinflammation [8], and selective degeneration of frontotemporal networks [9–12]. FTD accounts for 10–20% of dementia diagnoses [13–15], most commonly affecting people between the ages of 45 and 64 [16–20].

FTD encompasses three main clinical syndromes with different clinical characteristics: behavioral variant frontotemporal dementia (bvFTD), semantic variant primary progressive aphasia (svPPA), and non-fluent variant primary progressive aphasia (nfvPPA) [21]. bvFTD, the most common form of FTD, is characterized by early changes in behavior with social disinhibition, apathy, reduced sympathy and empathy, altered food preferences, and repetitive behavior [22]. These social and behavioral abnormalities, accompanied by a relative lack of amnestic deficits, are key distinguishing features of bvFTD [23]. svPPA is characterized by deficits in naming and semantic knowledge with preserved fluency [24–26]. nfvPPA is characterized by effortful, nonfluent, and agrammatic language deficits and impaired comprehension of complex sentences. Patients with nfvPPA often present with speech apraxia and may also have accompanying motor deficits, difficulty swallowing, and, eventually, mutism [27–29].

The prevalence of FTD in the US is around 60,000 cases, but this may be an underestimate since FTD is often misdiagnosed [18, 30]. Considering that the average age of onset is under 60, more than 10 years earlier than the average age of onset for Alzheimer’s disease, an FTD diagnosis carries high socioeconomic costs to patients and their families [31].

There are currently no FDA-approved treatments that slow or reverse progression of FTD. Pharmacologic interventions including serotonergic medications and antipsychotic agents, and non-pharmacologic interventions like behavior monitoring and speech therapy, are primarily used for symptom management and have limited effectiveness [32, 33].

Monogenic variants of FTD are uniquely amenable to personalized medicine approaches. Between 10 and 25% of FTD patients have autosomal dominant familial FTD [34–36]. Over 80% of patients with FTD caused by genetic mutations harbor heterozygous mutations in one of three genes: microtubule-associated protein tau (*MAPT*) [37, 38], chromosome 9 open reading frame 72 (*C9orf72*) [39–42], and progranulin (*GRN*) [2, 43, 44].

Unlike with *MAPT* and *C9orf72* mutations, FTD-related *GRN* mutations are almost exclusively loss-of-function, making progranulin replacement an attractive and conceptually straightforward therapeutic strategy. Most FTD-related *GRN* mutations result in premature stop codon insertion and nonsense-mediated RNA decay [2, 45]. Splice site and point mutations also result in progranulin haploinsufficiency by disrupting progranulin translation, maturation, or successful routing to the secretory pathway. The *GRN* A9D missense mutation disrupts the signal sequence of the progranulin peptide, thus preventing both its lysosomal localization and secretion into the extracellular space [44, 46–48]. Other missense and splice site mutations cause frameshifts or large genomic deletions that result in aberrant protein products ultimately destined for ER-associated degradation [49, 50]. Two pathogenic cysteine mutations, C521Y and C139R, do not cause progranulin haploinsufficiency but abolish progranulin’s neurotrophic effects in vitro and may impact progranulin’s proteolytic cleavage, which is necessary for production of functional granulins [51].

### Other Neurodegenerative Diseases

Loss-of-function progranulin mutations have been identified as a rare cause of dementia with Lewy bodies (DLB), a degenerative disorder associated with synuclein pathology [52]. Variation at the *GRN* locus is also associated with Alzheimer’s disease and limbic-predominant age-associated TDP-43 encephalopathy (LATE) [53, 54]. These observations suggest that the therapeutic strategies for raising progranulin levels discussed here may have applications for neurodegenerative diseases beyond FTD-*GRN.*

### Neuronal Ceroid Lipofuscinosis

FTD-related mutations in *GRN* are heterozygous and cause progranulin haploinsufficiency. In rare cases, homozygous or complex heterozygous mutations in *GRN* cause complete progranulin deficiency, which results in a lysosomal storage disorder called neuronal ceroid lipofuscinosis (NCL). The neuronal ceroid lipofuscinoses (NCLs) are a group of neurodegenerative lysosomal storage diseases associated with excessive accumulation of the waste pigment lipofuscin in neurons [55]. Early classification of the NCLs centered on the disease age of onset (infantile, late infantile, juvenile, and adult), but this is now supplanted by genetic classification based on discovery of loss-of-function mutations in genes that cause NCL: *PPT1*, *TPP1*, *CLN3*, *DNAJC5*, *CLN5*, *CLN6*, *MFSD8*, *CLN8*, *CTSD*, *GRN*, *ATP13A2*, *CTSF*, and *KCTD7* [56]. The NCLs are clinically heterogeneous, with disease onset ranging from infanthood to adulthood and wide variability in symptomology. Symptoms of the NCLs are primarily neurological; seizures, developmental regression, and motor deficits are seen in many cases. The retina is another prominent site of pathology for NCLs, and visual impairment is a common early sign for many cases of NCL [57].

NCL caused by progranulin deficiency, CLN11, was first described in 2012 in two siblings carrying homozygous *GRN* mutations (c.813_816del; p.Thr272Serfs*10) resulting in no detectable progranulin in blood or peripheral tissues [58]. Since the original report, a handful of individuals with homozygous or compound heterozygous loss-of-function *GRN* mutations have been identified, with most presenting with adult-onset NCL [59, 60].

Medical treatment for the NCLs is supportive, such as antiepileptic medications to treat disease-associated seizures. In 2017, the FDA approved a first-in-class enzyme replacement therapy for CLN2, which is caused by tripeptidylpeptidase 1 (*TPP1*) deficiency. This treatment, called cerliponase alfa (*Brineura*), is a recombinant TPP1 infused biweekly via intracerebroventricular catheter and dramatically slows the progression of disease [61]. Enzyme replacement therapy has been successful in alleviating phenotypes of other lysosomal storage diseases caused by loss-of-function mutations [62, 63]. Such success contributes to interest in progranulin replacement strategies as a therapeutic strategy, although progranulin is not an enzyme.

### Progranulin in Cancer

While insufficient progranulin causes neurodegenerative disease, increased progranulin is associated with cancer [64, 65]. These effects are likely related to progranulin’s trophic effects, discussed below. For example, high serum progranulin is a biomarker associated with poorer prognosis for several types of cancer including breast, lung, prostate, ovarian, and leukemia [66, 67]. There is also evidence that progranulin is more than just a biomarker but also a treatment target in cancer. Progranulin knockdown with antisense oligonucleotides or shRNA in malignant cells inhibits subsequent tumor growth upon implantation in xenograft models in vivo [68, 69]. Systemic treatment with progranulin-neutralizing antibodies also inhibits hepatocellular cancer cell growth in xenografts [70].

These observations suggest that promoting malignancy is a potential adverse effect of progranulin-raising therapies, although no studies to date have shown that progranulin-raising therapies are tumorigenic in animal models. Most of the data are from studies on malignancies in the periphery, which may suggest benefits of specifically brain-targeted progranulin therapies (although progranulin may have similar effect on glioblastoma cells [71]). Of course, with a progressive, fatal neurodegenerative disease, such longer-term risks would likely be outweighed if progranulin-increasing treatments have a clear impact on FTD-*GRN*.

## Progranulin Biology

Progranulin is a secreted and lysosome-resident glycoprotein comprising one partial and 7 full-length granulin domains, identifiable by their conserved double cysteine repeat motifs and separated by disordered linker polypeptides [72]. The granulins are believed to be the functional units of progranulin in the lysosome, as progranulin is rapidly cleaved into the granulins upon delivery to the lysosome [73]. Unlike the majority of soluble lysosomal proteins, which undergo sorting to the lysosome via addition of mannose-6-phosphate residues that are recognized by the mannose-6-phosphate receptor (M6PR) to drive lysosomal targeting [74], progranulin primarily uses non-canonical methods of reaching the lysosome. Sortilin is the primary internalization receptor for progranulin in many cell types, trafficking extracellular and intracellular progranulin to the lysosome through the interaction of sortilin’s beta-propeller domain with the three most C-terminal amino acids of progranulin, QLL in human progranulin and PLL in mouse progranulin [75, 76]. In addition, progranulin cooperatively transports with prosaposin, which undergoes lysosomal sorting through both M6PR and LDL receptor related protein 1 (LRP1) [77, 78].

Progranulin has numerous functions including lysosomal homeostasis, neuronal growth and maturation, neuroprotection, and immune regulation [79–83]. Progranulin regulates lysosomal biogenesis via acidification of lysosomes, and progranulin deficiency causes dramatic alterations of autophagic flux. Progranulin deficiency causes lipofuscinosis, a pathological accumulation of the lysosomal waste pigment lipofuscin, and aberrant activities of several lysosomal enzymes including cathepsin D and beta-glucocerebrosidase [77, 84–87]. Lysosome-associated membrane protein 1 (LAMP1), a marker of lysosomal membranes, also increases in the setting of progranulin deficiency, suggesting a general accumulation of lysosomes [88]. Progranulin’s neuroprotective effects also may be a result of its functions within the lysosome, as a lysosome-specific (i.e., non-secreted) progranulin is sufficient to ameliorate NMDA-induced excitotoxicity in primary cortical neurons [89]. As a neurotrophic factor, progranulin increases dendritic complexity in cellular models, and overexpression of progranulin stimulates axonal outgrowth in mouse models [77, 84–87]. In addition, delivery of recombinant progranulin to the cerebellum in a rat model of autism spectrum disorder increases synapse formation and reduces neuron loss [90]. Together, these data support a fundamental role of progranulin in neuronal growth and health. Progranulin deficiency, conversely, leads to synaptic dysfunction. Progranulin-knockout mice demonstrate impaired synaptic plasticity and reduced spine density in the CA1 region of the hippocampus [91], and patients with FTD-*GRN* exhibit thalamocortical circuit dysfunction recapitulated by progranulin-deficient mouse models [92, 93].

Multiple cell types produce progranulin, with neurons and microglia as the main sources [94]. In the brain under normal conditions, neuronal progranulin accounts for the majority of progranulin, as conditional deletion of a floxed progranulin allele using nestin-Cre leads to a > 50% reduction in progranulin, and using CaMKII-Cre leads to comparable reductions in brain regions where it is expressed [95, 96]. Microglia are another source of progranulin in the brain, and activated microglia upregulate progranulin expression [97]. Despite these two cell types producing significant amounts of progranulin, conditional deletion of either neuronal or microglial progranulin is insufficient to recapitulate the gliosis and lysosomal dysfunction that are seen in *Grn*^–/–^ mice [95, 96, 98]. Neurons can modulate microglial activity through progranulin, as progranulin secreted by neurons mediates microglial recruitment and phagocytotic activity [99]. Progranulin-knockout mice have reactive microgliosis and astrocytosis, and cultured microglia from these mice show increased synaptic pruning, cytotoxic, and pro-inflammatory activity [83, 88, 100, 101]. Progranulin’s immunomodulatory functions are not confined to the central nervous system, however. In asthma, progranulin inhibits neutrophil degranulation and stimulates regulatory T-cell proliferation, and, in patients with rheumatoid arthritis, progranulin levels are associated with disease activity [102, 103].

## Preclinical Models of Progranulin Deficiency

As a loss-of-function disorder, progranulin-knockout and knockin mice serve as useful models for FTD-GRN. These include both heterozygous and homozygous knockout mice, as well as knockin mice carrying the R493X nonsense mutation. Key similarities and differences between these models are summarized in Table 1.

**Table 1:** Comparison of selected features of progranulin mouse models

	***Grn***^**+/–**^	***Grn***^**–/–**^	***Grn***^**R493X/R493X**^	**Refs**
**General**				
Mechanism modeled	Progranulin haploinsufficiency, as in FTD-*GRN*	Full progranulin deficiency, as in CLN11	Full progranulin deficiency due to nonsense mutation	[105] [115] [126]
Survival	–	↓	↓	[100]
**Preclinical uses**				
For testing exogenous PGRN	+	+	+	[113] [88] [148] [117]
For increasing effects of endogenous PGRN encoded by intact allele	+	–	–	[136]
For testing NMD inhibitors	–	–	+	[126] [147]
**Behavior**				
Social dominance	↓	–	nd	[107]
Sociability	↓	↓	nd	[105]
Compulsive grooming	–	+	+	[101] [100]
**Lipid abnormalities**				
Gangliosidosis	–	↑	↑	[120]
BMP species	–	↓	↓	[120]
Glucosylsphingosine	–	↑	↑	[117]
**Pathology**				
Lipofuscinosis	–	↑	↑	[115] [126] [127]
Microgliosis	–	+	+	[115] [126] [127]
Astrocytosis	–	+	+	[100] [115] [127]
TDP43 Aggregates	–	+	+	[121–123, 126]
**Biomarkers**				
CSF NfL	nd	↑	nd	[117] [152]
TSPO µPET	nd	↑	nd	[152]

*nd* no data available

### Heterozygous Knockout Mice

Model validity is an important consideration for preclinical testing of neurotherapeutics [104]. Progranulin heterozygous knockout (*Grn*^+*/–*^) mice model the haploinsufficient etiology of FTD-*GRN* and recapitulate FTD-associated social behavioral deficits associated with selective degeneration of the salience network [105, 106]. *Grn*^+*/–*^ mice have an age-dependent losing phenotype in the tube test [107], a social dominance assay that depends on network activity between the medial prefrontal cortex (mPFC), amygdala, and mediodorsal nucleus of the thalamus [108, 109]. *Grn*^+*/–*^mice also recapitulate social withdrawal deficits characteristic of FTD-*GRN* as measured by the three-chamber sociability assay [105], a behavioral assay that measures a mouse’s preference for interacting with another mouse versus an inanimate object. Starting around 6 months of age, *Grn*^+*/–*^ mice spend significantly less time with another mouse relative to age-matched wild-type mice. This reduced sociability phenotype is preserved with age and is not a result of differences in olfactory perception of pheromones. *Grn*^+*/–*^ mice also have deficits in amygdala-dependent, hippocampus-independent classical cued fear conditioning.

The hippocampus is preserved during the early stages of FTD, and the pertinent lack of amnestic deficits in FTD is a key feature that differentiates FTD from Alzheimer’s disease. Interestingly *Grn*^+*/–*^ mice have normal hippocampal neuronal function as measured by extracellular field recordings of area CA1 of acute hippocampal slices and hippocampal-dependent spatial memory assays like the Morris water maze [105].

Other aspects of FTD clinical syndromes, such as impaired empathy or verbal communication, can be modeled in mice, but findings with these assays have not been reported in progranulin models. Of course, there are limits to how far complex human behavioral changes can be studied in mice. Taking an anatomical approach and focusing on dysfunction of conserved brain regions is one way to think about this issue. Many nodes in the salience network, the network most affected in FTD, are conserved between mice and humans [110]. As noted above, many of the phenotypes in *Grn*^+/–^ mice (and other FTD models, e.g., [111, 112]) are referable to dysfunction in these regions like the amygdala, thalamus, and mPFC.

An important limitation of *Grn*^+*/–*^ mice is that they do not exhibit most of the neuropathological characteristics of FTLD and lack translationally relevant biomarkers. No progranulin-deficient mouse models develop TDP-43 pathology to the extent seen in FTD-*GRN* patients. Neuron loss is not striking in *Grn*^+*/–*^ mice, although dendritic arbors of neurons in the prefrontal cortex are reduced with an age-related time course consistent with emergence of the social abnormalities [107]. The lysosomal pathology described below in *Grn*^*–/–*^ mice is much milder in *Grn*^+*/–*^ mice and develops only at late ages [113]. Unlike *Grn*^*–/–*^ mice, *Grn*^+*/–*^ mice do not exhibit increased age-dependent lipofuscinosis, microgliosis, or astrocytosis [105]. Demonstrating correction of social and other behavioral phenotypes, while useful, provides minimal information about underlying mechanistic pathways contributing to that correction. The lack of translationally relevant, quantifiable endpoints limits the ability to build PK/PD models or suggest translatable biomarkers for clinical evaluation in patients, and reinforces the need for additional CSF, plasma, and imaging-based studies in this model.

### Homozygous Knockout Mice

*Grn*^*–/–*^ mice model complete progranulin deficiency, which causes NCL in humans, so some consider these mice less of a model of FTD. However, *Grn*^*–/–*^ mice have several pathological features of FTD. Brains from patients with FTD-*GRN* have increased lipofuscinosis [114], aberrant elevations in levels and activity of hexosaminidase A, and concomitant decreases in levels and activity of beta-glucocerebrosidase [86]. These abnormalities are all modeled in *Grn*^*–/–*^ mice [86, 87, 115, 116]. *Grn*^*–/–*^ mice also have deficiency of bis(monoacylglycero)phosphate (BMP) species and glucosylsphingosine accumulation indicating abnormalities in sphingolipid metabolism [117–120]. Lipofuscinosis and lysosomal dyshomeostasis are apparent in *Grn*^*–/–*^ mice as early as 6 months of age, while deficits in sphingolipid metabolism start even earlier, at around 3 months of age. Although not to the extent seen in FTD-*GRN* patients, *Grn*^*–/–*^ mice develop some cytoplasmic TDP43 aggregates in areas of the thalamus from 12 to 25 months [121, 122] and in areas of the pons at 21 months [123]. In addition to lysosomal dysfunction, *Grn*^*–/–*^ mice also exhibit neuroinflammation, another key feature of FTD-*GRN* pathology. *Grn*^*–/–*^ mice have age-dependent upregulation of CD68, a lysosomal marker of microglial activation, and increases in microglial soma size in the thalamus [88, 95, 115, 119]. This inflammation is particularly prominent in the thalamus, where *Grn*^*–/–*^ microglia mediate increased pruning of inhibitory synapses [101]. *Grn*^*–/–*^ mice also have increased TNFα, an inflammatory cytokine, in the cortex and thalamus relative to age-matched *Grn*^+*/–*^ and *Grn*^+*/*+^ mice [105].These phenotypes may contribute to the decreased survival of *Grn*^*–/–*^ mice; in one study, the median survival of *Grn*^*–/–*^ mice was over 200 days less than that of *Grn*.^+*/*+^ mice [100].

Functional outcome measures in *Grn*^*–/–*^ mice have interesting and sometimes unexpected contrasts to *Grn*^+*/–*^ mice. Some behavioral abnormalities are similar in *Grn*^*–/–*^ and *Grn*^+*/–*^ mice, including decreased sociability and impaired amygdala-dependent fear conditioning [105]. Some outcomes are much more prominent in *Grn*^*–/–*^ mice, such as compulsive grooming [101, 124], which is also observed in tau FTD models [111] and models the compulsive behavior seen in many patients with FTD. The compulsive grooming in *Grn*^*–/–*^ mice has been associated with thalamic hyperactivity due to complement-mediated microglial synaptic pruning [101]. Similar thalamic hyperactivity is observed in *GRN* mutation carriers during presymptomatic stages [125]. Finally, some outcomes are actually less prominent in *Grn*^*–/–*^ mice than in *Grn*^+*/–*^ mice, most notably low social dominance which seems to be a unique feature of progranulin haploinsufficiency and not observed in *Grn*^*–/–*^ mice [107].

### R493X Knock-In Mice

Over 80% of FTD-causing *GRN* mutations introduce premature stop codons that result in truncated, nonfunctional progranulin protein. The most common *GRN* mutation is a premature stop codon replacement of arginine 493 (R493X) that results in a mutant mRNA product destined for cytoplasmic nonsense mediated decay. *Grn*^R493X/R493X^ mice have a nonsense mutation equivalent to human R493X (which in murine progranulin is R504X) that leads to nonsense-mediated decay and near-complete loss of progranulin. This model is thus uniquely amenable to preclinical testing of therapeutics targeting nonsense-mediated decay [126].

Heterozygous *Grn*^+/R493X^ mice have similar phenotypes to *Grn*^+*/–*^ mice, and homozygous *Grn*^R493X/R493X^ mice have similar phenotypes to *Grn*^*–/–*^ mice. Like *Grn*^*–/–*^ mice, *Grn*^R493X/R493X^ mice exhibit age-dependent thalamic microgliosis and lipofuscinosis as well as compulsive grooming and reduced survival relative to wild-type control mice [126, 127]. Also, like *Grn*^*–/–*^ mice, *Grn*^R493X/R493X^ mice have age-dependent accumulation of ganglioside species mono-sialylated GM1 and di-sialylated GD-3 in areas of the cortex, further supporting the idea that progranulin deficiency impairs sphingolipid degradation. The lipidomic profiles in brains of *Grn*^R493X/R493X^ mice resemble lipidomic changes in FTD-*GRN* [120]. Levels of monosialyated-GM1 and di-sialylated GD1 species are significantly elevated in brain tissue from patients with FTD-*GRN* but not in brain tissue from patients with sporadic-non-*GRN* FTD [120]. Finally*, Grn*^R493X/R493X^ mice also exhibit cytoplasmic accumulation of TDP43 and a dramatic reduction in synaptophysin [126].

In summary, *Grn*^+*/–*^ mice model progranulin haploinsufficiency, have relevant functional abnormalities but little neuropathology, and are useful for testing therapeutics intended to increase levels or enhance effects of progranulin produced from the intact allele. *Grn*^*–/–*^ mice model complete progranulin deficiency, have more robust neuropathology but lack some of the social behavioral abnormalities seen in *Grn*^+*/–*^ mice, and are useful for testing therapeutics that deliver exogenous progranulin. *Grn*^R493X^ mice model nonsense-mediated decay of progranulin, produce similar phenotypes to the knockout allele, and are useful for testing therapeutics that target this mechanism. Finally, all of these preclinical mouse models of FTD-*GRN* are also useful for evaluating safety concerns associated with progranulin-boosting therapeutics.

## Preclinical Testing of Progranulin Therapeutics

**Fig. 1:**
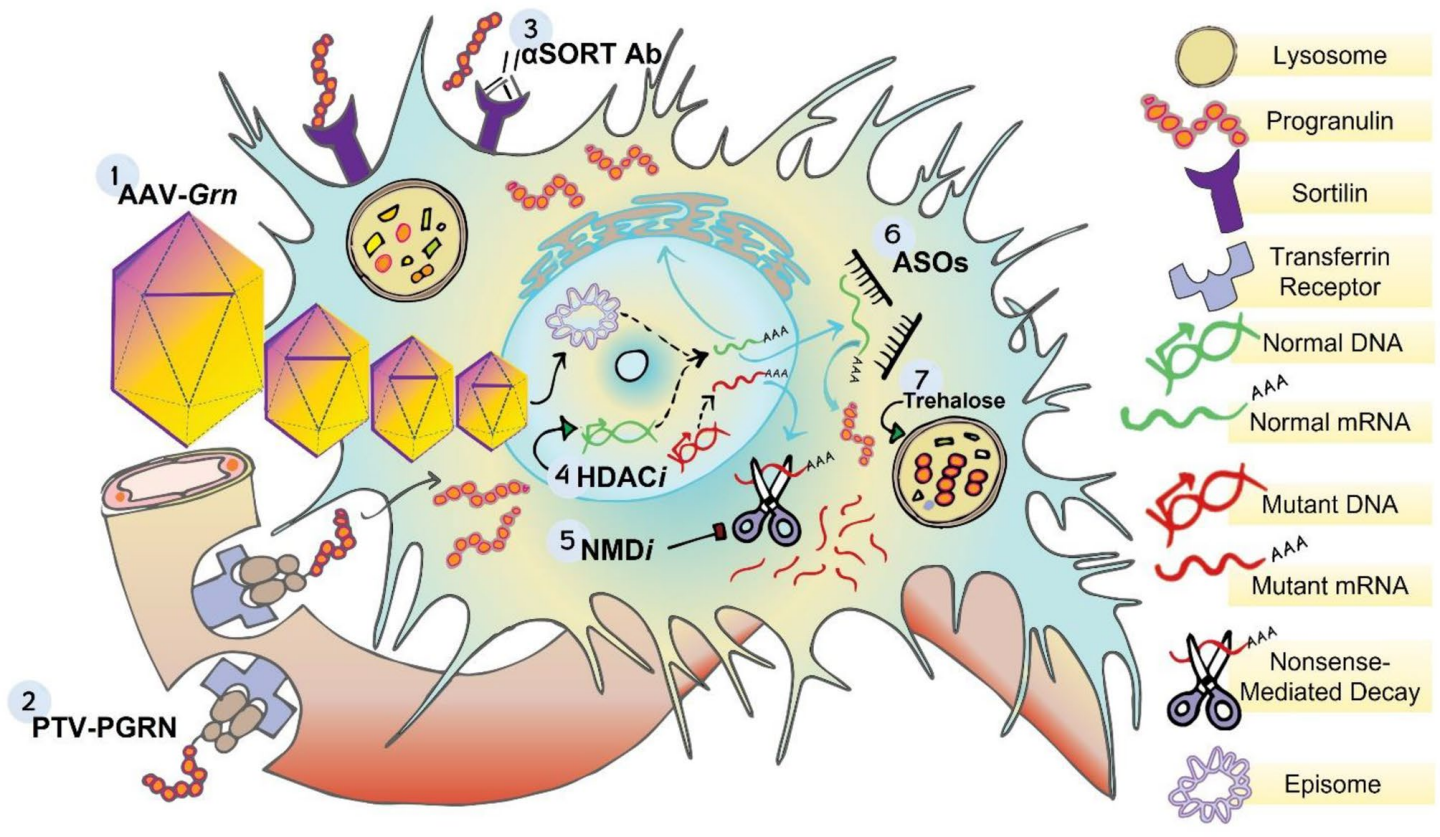
Preclinical interventions to optimize progranulin replacement. 1) AAV-progranulin gene therapy (*AAV-Grn*). Adeno-associated viral vectors (AAVs) consist of single stranded DNA encapsulated by icosahedral capsid proteins engineered to transduce specific cellular populations. Brain cells endocytose AAV capsids encoding progranulin. Once in the nucleus, AAV-derived progranulin DNA is uncoated into episomal DNA and subsequently transcribed by host machinery into progranulin mRNA. 2) Progranulin-conjugated protein transport vehicles (*PTV:PGRN*): protein transport vehicles (PTVs) are Fc domains engineered to bind human transferrin receptor that is highly expressed in the blood brain barrier. Progranulin can be fused to PTVs to be transported across the blood–brain barrier via transferrin receptor-mediated transcytosis. 3) Anti-sortilin antibodies (*α-SORT Abs*): sortilin mediates the uptake and intracellular degradation of progranulin. Sortilin-blocking antibodies reduce sortilin-mediated progranulin degradation, allowing for more peripheral and brain progranulin to accumulate in the extracellular space and enter cells via sortilin-independent pathways. 4) Histone de-acetylase inhibitors (*HDACis*): HDAC*i*s may boost progranulin levels by inducing progranulin transcription. Preclinical studies on the progranulin-boosting potential of HDAC*i*s are not as well studied in animal models of FTD-*GRN*, but one compound, FRM-0334, boosted hippocampal progranulin in wild-type mice. These data have not translated to clinical settings. 5) Inhibitors of nonsense mediated decay (*NMDis*): The majority of FTD-associated *GRN* mutations results in mutant progranulin mRNA products that are degraded by nonsense mediated decay machinery. Compounds that block nonsense mediated decay facilitate read-through of the mutant progranulin mRNA, thereby facilitating the translation of these species into progranulin protein. 6) Antisense oligonucleotides (*ASOs*): the microRNAs miR-29b, miR-107, and miR-659 are associated with decreases in progranulin translation. ASOs that block binding sites of miR-29b and miR-659 increase levels of normal progranulin mRNA, thereby facilitating the production of progranulin protein. 7) Trehalose: a disaccharide composed of two glucose molecules, was identified in a screen of small molecule autophagy-lysosome modulators. Oral administration of trehalose boosts levels of brain progranulin, suggesting that targeting autophagic flux is a potential approach for progranulin delivery

Several approaches to treating FTD-GRN have been evaluated in mouse models, a few of which have progressed to human clinical trials. These studies used a wide array of approaches targeting diverse mechanisms to increase progranulin (Fig. 1). We first consider indirect approaches to increase endogenous progranulin and then discuss two direct approaches to deliver exogenous progranulin. The indirect approaches highlighted here originated both from unbiased screens for compounds that could increase progranulin and from studies targeting known mechanisms of progranulin regulation.

### Hits from Unbiased Screens

One approach for identifying potential progranulin therapeutics is to screen libraries of compounds for the ability to increase progranulin levels. An early screen of approved drugs identified the histone deacetylase (HDAC) inhibitor suberoylanilide hydroxamic acid as an inducer of progranulin transcription [128]. Further work on HDAC inhibitors for progranulin was conducted mostly on human cell models rather than on rodent models that are the focus of this review [129]. The HDAC inhibitor FRM-0334 was tested in a phase 2 randomized clinical trial but showed no effect on progranulin levels in plasma or cerebrospinal fluid (CSF) [130].

Progranulin deficiency impairs autophagic flux, and significant effort has been devoted to finding endogenous and exogenous regulators of progranulin expression that may be druggable targets to increase progranulin levels. A screen of small-molecule autophagy-lysosome modulators identified several compounds that modify progranulin levels [131]. Trehalose, a disaccharide comprising two glucose molecules, was identified as a top hit, increasing both progranulin mRNA and protein levels [131]. In progranulin heterozygous knockout mice, oral administration of trehalose significantly increased progranulin levels in the brain [131].

### Nimodipine

Hypothesis-driven approaches based on the understanding of how progranulin is regulated have also been taken to identify progranulin therapeutics. Based on the idea that progranulin is regulated by calcium homeostasis, an early such approach tested several modulators of intracellular calcium and identified nimodipine, an FDA-approved calcium channel blocker that crosses the blood–brain barrier [132]. In preclinical studies in mouse models, nimodipine treatment for 2–3 weeks boosted hippocampal progranulin levels in *Grn*^+*/–*^ mice relative to vehicle-treated controls [133]. However, this preclinical data failed to translate in human *GRN* mutation carriers enrolled in an 8-week open label study on nimodipine. Oral nimodipine treatment did not have any effects on CSF or plasma PGRN levels and did not slow progressive brain atrophy in this cohort [133].

### Anti-Sortilin Antibodies

Sortilin, a transmembrane receptor in the VPS family, is a cell surface receptor for progranulin and mediates its neuronal uptake and intracellular degradation by trafficking to the lysosome. Genetic ablation of sortilin enhances circulating levels of progranulin in *Grn*^+*/–*^ mice, and small molecules that downregulate sortilin selectively increase extracellular progranulin levels in iPSC-derived neurons harboring the *GRN* S116X mutation [134]. Notably sortilin is not necessary for progranulin uptake, and extracellular progranulin can traffic to neuronal lysosomes via a sortilin-independent pathway that involves prosaposin-mediated entry via the M6PR/LRP1 pathway [78]. Thus, blocking the progranulin-sortilin interaction is a potential method to boost levels of progranulin in the extracellular space without compromising lysosomal localization of progranulin.

Multiple groups have developed anti-sortilin antibodies as a means of harnessing this mechanism to increase progranulin. These antibodies decrease sortilin levels on cultured cells and after systemic administration in mice, increase progranulin in plasma, CSF, and brain interstitial fluid [135]. One anti-sortilin antibody, AL001, has progressed to a phase 3 clinical trial (NCT04374136). The preclinical and phase 1 and 2 studies (NCT03636204 and NCT03987295) have not yet been published, but presentations at meetings have reported that intraperitoneal injection of AL001 increased brain and CSF progranulin levels in both *Grn*^+/+^ and *Grn*^+*/–*^ mice, as well as in healthy volunteers and FTD-*GRN* patients [136, 137].

### ASOs

Antisense oligonucleotide (ASO) therapies are becoming increasingly prevalent in the treatment of neurological disorders, notably including the relatively recent success of nusinersen for the treatment of spinal muscular atrophy in infants [138]. ASOs are oligonucleotides or analogs that can bind to several forms of RNA and alter its fate, classically targeting the RNA for degradation, inhibiting translation of mRNA, and modulating the splicing of the targeted RNA [138]. ASOs can also block microRNA binding to RNA, which is important because several microRNAs decrease progranulin translation: miR-29b, miR-107, and miR-659 [139–141]. One of these, miR-659, binds progranulin mRNA overlapping the rs5848 SNP, which modulates serum progranulin levels and is associated with both FTD and Alzheimer’s disease [141–145]. A recent preprint demonstrates that ASOs that block the binding sites of miR-29b and miR-659 increase progranulin levels, warranting further study on ASOs in models of progranulin haploinsufficiency [146]. ASOs can also inhibit nonsense-mediated decay by blocking the binding of proteins that recognize premature termination codons, and ASOs with this effect increase progranulin levels in cells from *Grn*^R493X/R493X^ mice [126].

### NMD Inhibitors

Nonsense-mediated decay (NMD) of mRNA occurs as a response to premature stop codons to prevent translation of nonfunctional proteins. Many progranulin mutations are nonsense mutations that lead to haploinsufficiency because of NMD. The most common disease-associated *GRN* mutation, R493X, leads to NMD of the mutant RNA in the *Grn*^R493X^ knockin mouse model [126]. NMD can be inhibited by “read-through” compounds that allow a tRNA to be incorporated into the protein at the nonsense stop codon. Such NMD inhibitors are being developed for a variety of genetic diseases that are caused by mutations inducing NMD. Inhibition of NMD with cycloheximide in *Grn*^R493X^ mice increased both progranulin mRNA and protein levels [126]. Similarly, the NMD inhibitor G418 increased readthrough of a progranulin R493X construct transduced into the brain with AAV [147]. In addition, the resulting truncated protein localized to lysosomes and recapitulated the functional effects of full-length progranulin [147]. Thus, for nonsense *GRN* mutations that result in NMD, read-through compounds may have promise, although no data has yet been published showing correction of downstream effects of progranulin deficiency by these compounds.

### PTV:PGRN

We now turn to two methods for direct delivery of exogenous progranulin to the central nervous system. Progranulin, like most large molecules, does not efficiently cross the blood–brain barrier (BBB), but advances in transport vehicle technology enabled the design of a delivery system that actively pumps progranulin through the BBB via receptor-mediated transcytosis. Human transferrin receptor (huTfR) is an endothelial protein that is highly abundant in the BBB and mediates the delivery of large molecules like Fc domain through the BBB into the brain. Protein transport vehicle conjugated progranulin (PTV:PGRN) consists of progranulin fused to an Fc domain that binds huTfR, leading to its delivery through the BBB [117]. PTV:PGRN, when delivered intraperitoneally, nearly doubled brain and peripheral progranulin levels in both *Grn*^+/+^ and *Grn*^*–/–*^ mice [117]. In as little as 72 h, PTV:PGRN restored lysosomal proteolysis and ameliorated production of reactive oxygen species in bone marrow–derived macrophages from *Grn*^–/–^ mice.

Peripheral PTV:PGRN treatment corrected BMP deficiency, a hallmark of dysregulated lipid metabolism, and GCase substrate accumulation in neurons, microglia, and astrocytes. PTV:PGRN also reduced lipofuscinosis, microgliosis, and astrocytosis in the thalamus, an area particularly vulnerable to lysosomal damage and neuroinflammation in the setting of total progranulin deficiency. Finally, PTV:PGRN also abrogated time-dependent increases in CSF levels of neurofilament light chain (NfL), a biomarker of neurodegeneration and axonal damage that is elevated both in patients with FTD-GRN and in mouse models of progranulin insufficiency. Altogether PTV:PGRN is a promising therapeutic for the FTD-GRN patient population and reinforces the therapeutic potential of progranulin replacement [117]. Denali, which developed PTV:PGRN and refers to it as DNL593, has planned an early-stage clinical trial (NCT05262023).

### Progranulin Gene Therapy

Unlike other progranulin-boosting strategies, AAV-progranulin (AAV-*Grn*) gene therapy could provide a one-time treatment yielding a long-lasting increase in CNS progranulin. Restoration of neuronal progranulin with AAV1-mouse-progranulin (AAV1-m*Grn*) corrected both neuronal and microglial phenotypes of progranulin deficiency in aged *Grn*^+*/–*^ and *Grn*^*–/–*^ mice [88, 113]. Delivery of AAV1-m*Grn* in the mPFC of 10- to 12-month-old *Grn*^+*/–*^ mice reversed mPFC-dependent social behavioral deficits and corrected elevated LAMP1 expression in the mPFC [113]. The therapeutic effects of AAV1-m*Grn* were not limited to the injection site. Delivery of AAV1-m*Grn* in the mPFC of 10- to 12-month-old *Grn*^*–/–*^ mice corrected lipofuscinosis and aberrant lysosomal enzyme activity in regions as distal as the thalamus, hippocampus, and motor cortex. Although AAV1 selectively transduces neurons, AAV1-m*Grn* also corrected microglial phenotypes of progranulin deficiency in the motor cortex, thalamus, and hippocampus [88].

Since progranulin is constitutively secreted, it has cross-correctional capacity in that AAV-derived progranulin is capable of being taken up and processed by non-transduced cells. For this reason, multiple labs have explored intracerebroventricular (ICV) delivery of AAV-progranulin packaged in capsids capable of transducing neurons, astroglia, and ependymal cells to maximize progranulin replacement. ICV delivery of human progranulin transgene product (h*Grn*) packaged in AAVhu68, an AAV9 variant that transduces neurons and astroglia, corrected lipofuscinosis, aberrant hexosaminidase activity, and microgliosis in *Grn*^*–/–*^ mice [148]. Interestingly, unlike AAVhu68, AAV1 can transduce both neurons and ependymal cells in brains of rhesus macaques. Intra–cisternamagna (ICM) delivery of AAV1-human progranulin in rhesus macaques results in significantly higher levels of CSF progranulin relative to AAVhu68-h*Grn* and AAV5-hu*Grn* [148].

Capsid serotype has a significant impact not only on the expression and efficacy of AAV-progranulin gene therapy but also its safety. AAVhu68 causes severe hepatotoxicity and neurotoxicity in non-human primates and proprioceptive deficits and ataxia in piglets [149]. ICV delivery of AAV9-hGrn caused almost complete hippocampal degeneration in *Grn*^*–/–*^ mice 6 months after treatment [150]. Hippocampal neurodegeneration was preceded by T-cell infiltration and perivascular cuffing, suggesting that the induction of a non-self-reaction by xenogeneic human progranulin transgene product was responsible. Hippocampal neurodegeneration has not been observed after transduction of murine progranulin in either *Grn*^–/–^ or *Grn*^+/+^ mice. Both AAVhu68 and AAV9 transduce glia in addition to neurons, which may exacerbate inflammatory responses to AAV delivery. While intraparenchymal delivery of AAV1-m*Grn* induces MHCII upregulation at the injection site in *Grn*^*–/–*^ mice, it has no detrimental effects on mPFC function and corrects neuroinflammation in distal regions [88], suggesting that restricting AAV-progranulin delivery to neurons may be safer and more effective than widespread cellular transduction.

Several companies have begun early-stage clinical trials with AAV-progranulin. Prevail Therapeutics’ PR006 is an AAV9-hGRN (NCT04408625) and Passage Bio’s PBFT02 is an AAV1-hGRN (NCT04747431). Both are administered by a single intracisternal magna injection.

## Conclusions

*GRN* mutations associated with FTD are loss-of-function, and nearly all cause progranulin haploinsufficiency. Progranulin replacement is a conceptually straightforward therapeutic approach for the FTD-*GRN* patient population, although as we have discussed there are several issues yet to be fully resolved. One issue is in which cell types and subcellular compartments progranulin levels must be restored. Because progranulin has diverse functions, understanding its cellular and subcellular biology is critical for the design of safe and effective progranulin-based therapies. Preclinical models will remain critical for addressing these questions.

Another issue is how best to raise progranulin. We have discussed a diverse suite of potential therapeutic strategies (Fig. 1), many of which are currently in clinical trials for FTD-*GRN*. Some of these strategies aim to improve endogenous progranulin levels/function by increasing expression of the intact allele (HDAC inhibitors), promoting translation of progranulin mRNA (ASOs), enabling read-through translation of the mutant allele (NMD inhibitors), or modifying progranulin trafficking to the lysosome (anti-sortilin antibodies). Other strategies aim to provide exogenous progranulin either by infusing a BBB-penetrating form (PTV:PGRN) or by gene therapy. Preclinical studies such as the ones we have outlined here were critical for the development of these programs and will continue to inform the next generation of therapeutics for FTD-*GRN*, including as a source of data about longer term adverse or off-target effects to de-risk safety concerns.

A third issue is when in the disease course these interventions should be delivered. Alzheimer’s disease clinical trials have indicated that disease-modifying therapies are more effective when delivered early, and, in theory, treatment of *GRN* mutation carriers could be initiated early in life. However, since the disease is generally asymptomatic until later in life, the cost and potential risks of early-life or long-term treatment may not be justified. Data from progranulin-deficient mouse models indicates that progranulin-boosting therapeutics can correct behavioral, biochemical, and neuropathological abnormalities even after onset, but initiating treatment after symptoms emerge would be limited by the fact that FTD-*GRN* progresses rapidly once it becomes symptomatic. In disease progression models, FTD-*GRN* had the fastest decline among the three major genetic etiologies of FTD [151]. The ideal timeframe for treatment initiation is probably a few years before symptom onset, and identifying patients at this stage will likely be enabled by plasma biomarkers. Levels of NfL begin to rise as early as 10 years before symptom onset in *GRN* mutation carriers, so longitudinally following plasma NfL from midlife may provide a means for guiding treatment decisions, initiating treatment once NfL starts to increase. Preclinical studies that identify progranulin-deficient mouse models with early increases in NfL have the potential to further inform the most optimal timing of progranulin-boosting treatments.

We are entering an exciting era for treatment of neurodegenerative diseases with an expanding toolbox of disease-modifying drugs, which hopefully will soon include progranulin-based therapeutics for FTD-*GRN*.

## Supplementary Information

Below is the link to the electronic supplementary material.

Supplementary file1 (PDF 499 kb)

Supplementary file2 (PDF 499 kb)

Supplementary file3 (PDF 498 kb)

## References

[1] Alonso ADC, Mederlyova A, Novak M, Grundke-Iqbal I, Iqbal K (2004). Promotion of hyperphosphorylation by frontotemporal dementia tau mutations. J Biol Chem.

[2] Baker M, Mackenzie IR, Pickering-Brown SM (2006). Mutations in progranulin cause tau-negative frontotemporal dementia linked to chromosome 17. Nature.

[3] Cairns NJ, Bigio EH, Mackenzie IR (2007). Neuropathologic diagnostic and nosologic criteria for frontotemporal lobar degeneration: consensus of the Consortium for Frontotemporal Lobar Degeneration. Acta Neuropathol.

[4] Brettschneider J, Del Tredici K, Irwin DJ (2014). Sequential distribution of pTDP-43 pathology in behavioral variant frontotemporal dementia (bvFTD). Acta Neuropathol.

[5] Mackenzie IR (2007). The neuropathology and clinical phenotype of FTD with *progranulin* mutations. Acta Neuropathol.

[6] Neumann M, Mackenzie IR, Cairns NJ (2007). TDP-43 in the ubiquitin pathology of frontotemporal dementia with VCP gene mutations. J Neuropathol Exp Neurol.

[7] Mackenzie IR, Baborie A, Pickering-Brown S (2006). Heterogeneity of ubiquitin pathology in frontotemporal lobar degeneration: classification and relation to clinical phenotype. Acta Neuropathol.

[8] Kim M-J, McGwier M, Jenko KJ (2019). Neuroinflammation in frontotemporal lobar degeneration revealed by (11) C-PBR28 PET. Ann Clin Transl Neurol.

[9] Seeley WW, Crawford RK, Zhou J, Miller BL, Greicius MD (2009). Neurodegenerative diseases target large-scale human brain networks. Neuron.

[10] Dopper EG, Rombouts SA, Jiskoot LC (2014). Structural and functional brain connectivity in presymptomatic familial frontotemporal dementia. Neurology.

[11] Kim EJ, Sidhu M, Gaus SE (2012). Selective frontoinsular von Economo neuron and fork cell loss in early behavioral variant frontotemporal dementia. Cereb Cortex.

[12] Zhou J, Greicius MD, Gennatas ED (2010). Divergent network connectivity changes in behavioural variant frontotemporal dementia and Alzheimer's disease. Brain.

[13] Coyle-Gilchrist IT, Dick KM, Patterson K (2016). Prevalence, characteristics, and survival of frontotemporal lobar degeneration syndromes. Neurology.

[14] Knopman DS, Roberts RO (2011). Estimating the number of persons with frontotemporal lobar degeneration in the US population. J Mol Neurosci.

[15] Ratnavalli E, Brayne C, Dawson K, Hodges JR (2002). The prevalence of frontotemporal dementia. Neurology.

[16] Chiu WZ, Kaat LD, Seelaar H (2010). Survival in progressive supranuclear palsy and frontotemporal dementia. J Neurol Neurosurg Psychiatry.

[17] Seeley WW, Bauer AM, Miller BL (2005). The natural history of temporal variant frontotemporal dementia. Neurology.

[18] Rosso SM, Donker Kaat L, Baks T (2003). Frontotemporal dementia in The Netherlands: patient characteristics and prevalence estimates from a population-based study. Brain.

[19] Onyike CU, Diehl-Schmid J (2013). The epidemiology of frontotemporal dementia. Int Rev Psychiatry.

[20] Roberson ED, Hesse JH, Rose KD (2005). Frontotemporal dementia progresses to death faster than Alzheimer disease. Neurology.

[21] Ljubenkov PA, Miller BL (2016). A clinical guide to frontotemporal dementias. Focus.

[22] Karageorgiou E, Miller BL (2014). Frontotemporal lobar degeneration: a clinical approach. Semin Neurol.

[23] Rascovsky K, Hodges JR, Kipps CM (2007). Diagnostic criteria for the behavioral variant of frontotemporal dementia (bvFTD): current limitations and future directions. Alzheimer Dis Assoc Disord.

[24] Hodges JR, Patterson K (2007). Semantic dementia: a unique clinicopathological syndrome. Lancet Neurol.

[25] Laisney M, Matuszewski V, Mezenge F (2009). The underlying mechanisms of verbal fluency deficit in frontotemporal dementia and semantic dementia. J Neurol.

[26] Mummery CJ, Patterson K, Price CJ, Ashburner J, Frackowiak RS, Hodges JR (2000). A voxel-based morphometry study of semantic dementia: relationship between temporal lobe atrophy and semantic memory. Ann Neurol.

[27] Caso F, Mandelli ML, Henry M (2014). In vivo signatures of nonfluent/agrammatic primary progressive aphasia caused by FTLD pathology. Neurology.

[28] Gorno-Tempini ML, Hillis AE, Weintraub S (2011). Classification of primary progressive aphasia and its variants. Neurology.

[29] Grossman M (2010). Primary progressive aphasia: clinicopathological correlations. Nat Rev Neurol.

[30] Zapata-Restrepo L, Rivas J, Miranda C, et al. The psychiatric misdiagnosis of behavioral variant frontotemporal dementia in a Colombian sample. Front Neurol. 2021;12. 10.3389/fneur.2021.729381 PMC8634474 34867716

[31] Galvin JE, Howard DH, Denny SS, Dickinson S, Tatton N (2017). The social and economic burden of frontotemporal degeneration. Neurology.

[32] Tsai RM, Boxer AL (2014). Treatment of frontotemporal dementia. Curr Treat Options Neurol.

[33] Casaletto KB, Staffaroni AM, Wolf A (2020). Active lifestyles moderate clinical outcomes in autosomal dominant frontotemporal degeneration. Alzheimers Dement.

[34] Roberson ED (2006). Frontotemporal dementia. Curr Neurol Neurosci Rep.

[35] Chow TW, Miller BL, Hayashi VN, Geschwind DH (1999). Inheritance of frontotemporal dementia. Arch Neurol.

[36] Greaves CV, Rohrer JD (2019). An update on genetic frontotemporal dementia. J Neurol.

[37] van Swieten J, Spillantini MG (2007). Hereditary frontotemporal dementia caused by *tau* gene mutations. Brain Pathol.

[38] Cruts M, Rademakers R, Gijselinck I (2005). Genomic architecture of human 17q21 linked to frontotemporal dementia uncovers a highly homologous family of low-copy repeats in the tau region. Hum Mol Genet.

[39] Almeida S, Gascon E, Tran H (2013). Modeling key pathological features of frontotemporal dementia with C9ORF72 repeat expansion in iPSC-derived human neurons. Acta Neuropathol.

[40] Dobson-Stone C, Hallupp M, Loy CT (2013). C9ORF72 repeat expansion in Australian and Spanish frontotemporal dementia patients. PLoS ONE.

[41] DeJesus-Hernandez M, Mackenzie IR, Boeve BF (2011). Expanded GGGGCC hexanucleotide repeat in noncoding region of *C9ORF72* causes chromosome 9p-linked FTD and ALS. Neuron.

[42] Devenney E, Hornberger M, Irish M (2014). Frontotemporal dementia associated with the C9ORF72 mutation: a unique clinical profile. JAMA Neurol.

[43] Gijselinck I, van der Zee J, Engelborghs S (2008). Progranulin locus deletion in frontotemporal dementia. Hum Mutat.

[44] Mukherjee O, Wang J, Gitcho M (2008). Molecular characterization of novel progranulin (*GRN*) mutations in frontotemporal dementia. Hum Mutat.

[45] Cruts M, Gijselinck I, van der Zee J (2006). Null mutations in progranulin cause ubiquitin-positive frontotemporal dementia linked to chromosome 17q21. Nature.

[46] Gass J, Cannon A, Mackenzie IR (2006). Mutations in progranulin are a major cause of ubiquitin-positive frontotemporal lobar degeneration. Hum Mol Genet.

[47] Le Ber I, van der Zee J, Hannequin D (2007). Progranulin null mutations in both sporadic and familial frontotemporal dementia. Hum Mutat.

[48] Shankaran SS, Capell A, Hruscha AT (2008). Missense mutations in the progranulin gene linked to frontotemporal lobar degeneration with ubiquitin-immunoreactive inclusions reduce progranulin production and secretion. J Biol Chem.

[49] Bronner IF, Rizzu P, Seelaar H (2007). Progranulin mutations in Dutch familial frontotemporal lobar degeneration. Eur J Hum Genet.

[50] Hosaka T, Ishii K, Miura T (2017). A novel frameshift GRN mutation results in frontotemporal lobar degeneration with a distinct clinical phenotype in two siblings: case report and literature review. BMC Neurol.

[51] Wang J, Van Damme P, Cruchaga C (2010). Pathogenic cysteine mutations affect progranulin function and production of mature granulins. J Neurochem.

[52] Reho P, Koga S, Shah Z (2022). *GRN* Mutations are Associated with Lewy body dementia. Mov Disord.

[53] Perry DC, Lehmann M, Yokoyama JS (2013). Progranulin mutations as risk factors for Alzheimer disease. JAMA Neurol.

[54] Dugan AJ, Nelson PT, Katsumata Y (2021). Analysis of genes (*TMEM106B, GRN, ABCC9, KCNMB2, and APOE*) implicated in risk for LATE-NC and hippocampal sclerosis provides pathogenetic insights: a retrospective genetic association study. Acta Neuropathol Commun.

[55] Mole SE, Anderson G, Band HA (2019). Clinical challenges and future therapeutic approaches for neuronal ceroid lipofuscinosis. Lancet Neurol.

[56] Cotman SL, Karaa A, Staropoli JF, Sims KB (2013). Neuronal ceroid lipofuscinosis: impact of recent genetic advances and expansion of the clinicopathologic spectrum. Curr Neurol Neurosci Rep.

[57] Weleber RG (1998). The dystrophic retina in multisystem disorders: the electroretinogram in neuronal ceroid lipofuscinoses. Eye.

[58] Smith KR, Damiano J, Franceschetti S (2012). Strikingly different clinicopathological phenotypes determined by progranulin-mutation dosage. Am J Hum Genet.

[59] Kamate M, Detroja M, Hattiholi V (2019). Neuronal ceroid lipofuscinosis type-11 in an adolescent. Brain Dev.

[60] Huin V, Barbier M, Bottani A (2020). Homozygous GRN mutations: new phenotypes and new insights into pathological and molecular mechanisms. Brain.

[61] Schulz A, Ajayi T, Specchio N (2018). Study of intraventricular cerliponase alfa for CLN2 disease. N Engl J Med.

[62] Zimran A, Elstein D (2014). Management of Gaucher disease: enzyme replacement therapy. Pediatr Endocrinol Rev.

[63] Alegra T, Vairo F, de Souza MV, Krug BC, Schwartz IV (2012). Enzyme replacement therapy for Fabry disease: a systematic review and meta-analysis. Genet Mol Biol.

[64] Arechavaleta-Velasco F, Perez-Juarez CE, Gerton GL, Diaz-Cueto L (2017). Progranulin and its biological effects in cancer. Med Oncol.

[65] Zhou C, Huang Y, Wu J (2021). A narrative review of multiple mechanisms of progranulin in cancer: a potential target for anti-cancer therapy. Transl Cancer Res.

[66] Tkaczuk KR, Yue B, Zhan M (2011). Increased circulating level of the survival factor GP88 (progranulin) in the serum of breast cancer patients when compared to healthy subjects. Breast Cancer (Auckl).

[67] Koo DH, Park CY, Lee ES, Ro J, Oh SW (2012). Progranulin as a prognostic biomarker for breast cancer recurrence in patients who had hormone receptor-positive tumors: a cohort study. PLoS ONE.

[68] Buraschi S, Xu SQ, Stefanello M (2016). Suppression of progranulin expression inhibits bladder cancer growth and sensitizes cancer cells to cisplatin. Oncotarget.

[69] Zhang H, Serrero G (1998). Inhibition of tumorigenicity of the teratoma PC cell line by transfection with antisense cDNA for PC cell-derived growth factor (PCDGF, epithelin/granulin precursor). Proc Natl Acad Sci U S A.

[70] Wong NC, Cheung PF, Yip CW (2014). Antibody against granulin-epithelin precursor sensitizes hepatocellular carcinoma to chemotherapeutic agents. Mol Cancer Ther.

[71] Bandey I, Chiou SH, Huang AP, Tsai JC, Tu PH (2015). Progranulin promotes temozolomide resistance of glioblastoma by orchestrating DNA repair and tumor stemness. Oncogene.

[72] Tolkatchev D, Malik S, Vinogradova A (2008). Structure dissection of human progranulin identifies well-folded granulin/epithelin modules with unique functional activities. Protein Sci.

[73] Holler CJ, Taylor G, Deng Q, Kukar T. Intracellular proteolysis of progranulin generates stable, lysosomal granulins that are haploinsufficient in patients with frontotemporal dementia caused by GRN mutations. eNeuro. 2017;4. 10.1523/ENEURO.0100-17.2017 PMC5562298 28828399

[74] Griffiths G, Hoflack B, Simons K, Mellman I, Kornfeld S (1988). The mannose 6-phosphate receptor and the biogenesis of lysosomes. Cell.

[75] Hu F, Padukkavidana T, Vægter CB (2010). Sortilin-mediated endocytosis determines levels of the frontotemporal dementia protein, progranulin. Neuron.

[76] Zheng Y, Brady OA, Meng PS, Mao Y, Hu F (2011). C-terminus of progranulin interacts with the beta-propeller region of sortilin to regulate progranulin trafficking. PLoS ONE.

[77] Valdez C, Ysselstein D, Young TJ, Zheng J, Krainc D (2020). Progranulin mutations result in impaired processing of prosaposin and reduced glucocerebrosidase activity. Hum Mol Genet.

[78] Zhou X, Sun L, Bastos de Oliveira F (2015). Prosaposin facilitates sortilin-independent lysosomal trafficking of progranulin. J Cell Biol.

[79] Evers BM, Rodriguez-Navas C, Tesla RJ (2017). Lipidomic and transcriptomic basis of lysosomal dysfunction in progranulin deficiency. Cell Rep.

[80] Hyung S, Im SK, Lee BY (2019). Dedifferentiated Schwann cells secrete progranulin that enhances the survival and axon growth of motor neurons. Glia.

[81] Ryan CL, Baranowski DC, Chitramuthu BP (2009). Progranulin is expressed within motor neurons and promotes neuronal cell survival. BMC Neurosci.

[82] Tapia L, Milnerwood A, Guo A (2011). Progranulin deficiency decreases gross neural connectivity but enhances transmission at individual synapses. J Neurosci.

[83] Yin F, Banerjee R, Thomas B (2010). Exaggerated inflammation, impaired host defense, and neuropathology in progranulin-deficient mice. J Exp Med.

[84] Valdez C, Wong YC, Schwake M, Bu G, Wszolek ZK, Krainc D (2017). Progranulin-mediated deficiency of cathepsin D results in FTD and NCL-like phenotypes in neurons derived from FTD patients. Hum Mol Genet.

[85] Zhou X, Paushter DH, Feng T, Pardon CM, Mendoza CS, Hu F (2017). Regulation of cathepsin D activity by the FTLD protein progranulin. Acta Neuropathol.

[86] Arrant AE, Roth JR, Boyle NR (2019). Impaired beta-glucocerebrosidase activity and processing in frontotemporal dementia due to progranulin mutations. Acta Neuropathol Commun.

[87] Zhou X, Paushter DH, Pagan MD (2019). Progranulin deficiency leads to reduced glucocerebrosidase activity. PLoS ONE.

[88] Arrant AE, Onyilo VC, Unger DE, Roberson ED (2018). Progranulin gene therapy improves lysosomal dysfunction and microglial pathology associated with frontotemporal dementia and neuronal ceroid lipofuscinosis. J Neurosci.

[89] Davis SE, Roth JR, Aljabi Q (2021). Delivering progranulin to neuronal lysosomes protects against excitotoxicity. J Biol Chem.

[90] Wang L, Chen J, Hu Y (2022). Progranulin improves neural development via the PI3K/Akt/GSK-3β pathway in the cerebellum of a VPA-induced rat model of ASD. Transl Psychiatry.

[91] Petkau TL, Neal SJ, Milnerwood A (2012). Synaptic dysfunction in progranulin-deficient mice. Neurobiol Dis.

[92] Beel S, Moisse M, Damme M (2017). Progranulin functions as a cathepsin D chaperone to stimulate axonal outgrowth in vivo. Hum Mol Genet.

[93] Longhena F, Zaltieri M, Grigoletto J (2017). Depletion of progranulin reduces GluN2B-containing NMDA receptor density, tau phosphorylation, and dendritic arborization in mouse primary cortical neurons. J Pharmacol Exp Ther.

[94] Petkau TL, Neal SJ, Orban PC (2010). Progranulin expression in the developing and adult murine brain. J Comp Neurol.

[95] Arrant AE, Filiano AJ, Patel AR (2018). Reduction of microglial progranulin does not exacerbate pathology or behavioral deficits in neuronal progranulin-insufficient mice. Neurobiol Dis.

[96] Petkau TL, Blanco J, Leavitt BR (2017). Conditional loss of progranulin in neurons is not sufficient to cause neuronal ceroid lipofuscinosis-like neuropathology in mice. Neurobiol Dis.

[97] Dong T, Tejwani L, Jung Y, et al. Microglia regulate brain progranulin levels through the endocytosis/lysosomal pathway. JCI Insight. 2021;6. 10.1172/jci.insight.136147 PMC8663778 34618685

[98] Petkau TL, Kosior N, de Asis K, Connolly C, Leavitt BR (2017). Selective depletion of microglial progranulin in mice is not sufficient to cause neuronal ceroid lipofuscinosis or neuroinflammation. J Neuroinflammation.

[99] Pickford F, Marcus J, Camargo LM (2011). Progranulin is a chemoattractant for microglia and stimulates their endocytic activity. Am J Pathol.

[100] Ghoshal N, Dearborn JT, Wozniak DF, Cairns NJ (2012). Core features of frontotemporal dementia recapitulated in progranulin knockout mice. Neurobiol Dis.

[101] Lui H, Zhang J, Makinson SR (2016). Progranulin deficiency promotes circuit-specific synaptic pruning by microglia via complement activation. Cell.

[102] Pogonowska M, Poniatowski ŁA, Wawrzyniak A, Królikowska K, Kalicki B (2019). The role of progranulin (PGRN) in the modulation of anti-inflammatory response in asthma. Cent Eur J Immunol.

[103] Andrés Cerezo L, Kuklová M, Hulejová H, et al. Progranulin is associated with disease activity in patients with rheumatoid arthritis. Mediators Inflamm. 2015;2015. 10.1155/2015/740357 PMC4539115 26339140

[104] Tadenev ALD, Burgess RW (2019). Model validity for preclinical studies in precision medicine: precisely how precise do we need to be?. Mamm Genome.

[105] Filiano AJ, Martens LH, Young AH (2013). Dissociation of frontotemporal dementia-related deficits and neuroinflammation in progranulin haploinsufficient mice. J Neurosci.

[106] Seeley WW (2010). Anterior insula degeneration in frontotemporal dementia. Brain Struct Funct.

[107] Arrant AE, Filiano AJ, Warmus BA, Hall AM, Roberson ED (2016). Progranulin haploinsufficiency causes biphasic social dominance abnormalities in the tube test. Genes Brain Behav.

[108] Zhou T, Zhu H, Fan Z (2017). History of winning remodels thalamo-PFC circuit to reinforce social dominance. Science.

[109] Wang F, Zhu J, Zhu H, Zhang Q, Lin Z, Hu H (2011). Bidirectional control of social hierarchy by synaptic efficacy in medial prefrontal cortex. Science.

[110] Roberson ED (2012). Mouse models of frontotemporal dementia. Ann Neurol.

[111] Warmus BA, Sekar DR, McCutchen E (2014). Tau-mediated NMDA receptor impairment underlies dysfunction of a selectively vulnerable network in a mouse model of frontotemporal dementia. J Neurosci.

[112] Gascon E, Lynch K, Ruan H (2014). Alterations in microRNA-124 and AMPA receptors contribute to social behavioral deficits in frontotemporal dementia. Nat Med.

[113] Arrant AE, Filiano AJ, Unger DE, Young AH, Roberson ED (2017). Restoring neuronal progranulin reverses deficits in a mouse model of frontotemporal dementia. Brain.

[114] Ward ME, Chen R, Huang HY, et al. Individuals with progranulin haploinsufficiency exhibit features of neuronal ceroid lipofuscinosis. Sci Transl Med. 2017;9. 10.1126/scitranslmed.aah5642 PMC5526610 28404863

[115] Ahmed Z, Sheng H, Xu YF (2010). Accelerated lipofuscinosis and ubiquitination in granulin knockout mice suggest a role for progranulin in successful aging. Am J Pathol.

[116] Arrant AE, Nicholson AM, Zhou X, Rademakers R, Roberson ED (2018). Partial Tmem106b reduction does not correct abnormalities due to progranulin haploinsufficiency. Mol Neurodegener.

[117] Logan T, Simon MJ, Rana A (2021). Rescue of a lysosomal storage disorder caused by *Grn* loss of function with a brain penetrant progranulin biologic. Cell.

[118] Huang M, Modeste E, Dammer E (2020). Network analysis of the progranulin-deficient mouse brain proteome reveals pathogenic mechanisms shared in human frontotemporal dementia caused by GRN mutations. Acta Neuropathol Commun.

[119] Gotzl JK, Colombo AV, Fellerer K (2018). Early lysosomal maturation deficits in microglia triggers enhanced lysosomal activity in other brain cells of progranulin knockout mice. Mol Neurodegener.

[120] Boland S, Swarup S, Ambaw YA (2022). Deficiency of the frontotemporal dementia gene *GRN* results in gangliosidosis. Nat Commun.

[121] Tanaka Y, Chambers JK, Matsuwaki T, Yamanouchi K, Nishihara M (2014). Possible involvement of lysosomal dysfunction in pathological changes of the brain in aged progranulin-deficient mice. Acta Neuropathol Commun.

[122] Zhang J, Velmeshev D, Hashimoto K (2020). Neurotoxic microglia promote TDP-43 proteinopathy in progranulin deficiency. Nature.

[123] Wu Y, Shao W, Todd TW (2021). Microglial lysosome dysfunction contributes to white matter pathology and TDP-43 proteinopathy in GRN-associated FTD. Cell Rep.

[124] Krabbe G, Minami SS, Etchegaray JI (2017). Microglial NFκB-TNFα hyperactivation induces obsessive-compulsive behavior in mouse models of progranulin-deficient frontotemporal dementia. Proc Natl Acad Sci U S A.

[125] Lee SE, Sias AC, Kosik EL (2019). Thalamo-cortical network hyperconnectivity in preclinical progranulin mutation carriers. Neuroimage Clin.

[126] Nguyen AD, Nguyen TA, Zhang J (2018). Murine knockin model for progranulin-deficient frontotemporal dementia with nonsense-mediated mRNA decay. Proc Natl Acad Sci U S A.

[127] Frew J, Nygaard HB (2021). Neuropathological and behavioral characterization of aged Grn R493X progranulin-deficient frontotemporal dementia knockin mice. Acta Neuropathol Commun.

[128] Cenik B, Sephton CF, Dewey CM (2011). Suberoylanilide hydroxamic acid (vorinostat) up-regulates progranulin transcription: rational therapeutic approach to frontotemporal dementia. J Biol Chem.

[129] Moreno-Yruela C, Fass DM, Cheng C, Herz J, Olsen CA, Haggarty SJ (2019). Kinetic tuning of HDAC inhibitors affords potent inducers of progranulin expression. ACS Chem Neurosci.

[130] Ljubenkov PA, Edwards L, Iaccarino L (2021). Effect of the histone deacetylase inhibitor FRM-0334 on progranulin levels in patients with progranulin gene haploinsufficiency: a randomized clinical trial. JAMA Netw Open.

[131] Holler CJ, Taylor G, McEachin ZT (2016). Trehalose upregulates progranulin expression in human and mouse models of GRN haploinsufficiency: a novel therapeutic lead to treat frontotemporal dementia. Mol Neurodegener.

[132] Tomassoni D, Lanari A, Silvestrelli G, Traini E, Amenta F (2008). Nimodipine and its use in cerebrovascular disease: evidence from recent preclinical and controlled clinical studies. Clin Exp Hypertens.

[133] Sha SJ, Miller ZA, Min SW (2017). An 8-week, open-label, dose-finding study of nimodipine for the treatment of progranulin insufficiency from GRN gene mutations. Alzheimers Dement (N Y).

[134] Almeida S, Zhang Z, Coppola G (2012). Induced pluripotent stem cell models of progranulin-deficient frontotemporal dementia uncover specific reversible neuronal defects. Cell Rep.

[135] Miyakawa S, Sakuma H, Warude D (2020). Anti-sortilin1 antibody up-regulates progranulin via sortilin1 down-regulation. Front Neurosci.

[136] Rosenthal A, Schwabe T, Kurnellas M, inventors; Alector LLC, assignee. Anti-sortilin antibodies and methods of use thereof. United States patent US10087255B2. 2018.

[137] Shugart J. AL001 boosts progranulin. Does it slow frontotemporal dementia? AlzForum [Internet]. 2021. Available from: https://www.alzforum.org/news/conference-coverage/al001-boosts-progranulin-does-it-slow-frontotemporal-dementia. Accessed 10 Jan 2022

[138] Hoy SM (2017). Nusinersen: first global approval. Drugs.

[139] Jiao J, Herl LD, Farese RV, Gao FB (2010). MicroRNA-29b regulates the expression level of human progranulin, a secreted glycoprotein implicated in frontotemporal dementia. PLoS ONE.

[140] Wang WX, Wilfred BR, Madathil SK (2010). miR-107 regulates granulin/progranulin with implications for traumatic brain injury and neurodegenerative disease. Am J Pathol.

[141] Rademakers R, Eriksen JL, Baker M (2008). Common variation in the miR-659 binding-site of GRN is a major risk factor for TDP43-positive frontotemporal dementia. Hum Mol Genet.

[142] Lee MJ, Chen TF, Cheng TW, Chiu MJ (2011). rs5848 variant of progranulin gene is a risk of Alzheimer's disease in the Taiwanese population. Neurodegener Dis.

[143] Sheng J, Su L, Xu Z, Chen G (2014). Progranulin polymorphism rs5848 is associated with increased risk of Alzheimer's disease. Gene.

[144] Fenoglio C, Galimberti D, Cortini F (2009). Rs5848 variant influences GRN mRNA levels in brain and peripheral mononuclear cells in patients with Alzheimer's disease. J Alzheimers Dis.

[145] Hsiung G-YR, Fok A, Feldman HH, Rademakers R, Mackenzie IRA (2011). rs5848 polymorphism and serum progranulin level. J Neurol Sci.

[146] Aggarwal G, Banerjee S, Jones SA, et al. Antisense oligonucleotides targeting the miR-29b binding site in the *GRN* mRNA increase progranulin translation. bioRxiv. 2022:2022.01.12.476053. 10.1016/j.jbc.2023.105475 PMC10755782 37981208

[147] Frew J, Baradaran-Heravi A, Balgi AD (2020). Premature termination codon readthrough upregulates progranulin expression and improves lysosomal function in preclinical models of GRN deficiency. Mol Neurodegener.

[148] Hinderer C, Miller R, Dyer C (2020). Adeno-associated virus serotype 1-based gene therapy for FTD caused by GRN mutations. Ann Clin Transl Neurol.

[149] Hinderer C, Katz N, Buza EL (2018). Severe toxicity in nonhuman primates and piglets following high-dose intravenous administration of an adeno-associated virus vector expressing human SMN. Hum Gene Ther.

[150] Amado DA, Rieders JM, Diatta F (2019). AAV-mediated progranulin delivery to a mouse model of progranulin deficiency causes T cell-mediated toxicity. Mol Ther.

[151] Staffaroni AM, Quintana M, Wendelberger B (2022). Temporal order of clinical and biomarker changes in familial frontotemporal dementia. Nat Med.

[152] Reifschneider A, Robinson S, van Lengerich B (2022). Loss of TREM2 rescues hyperactivation of microglia, but not lysosomal deficits and neurotoxicity in models of progranulin deficiency. EMBO J.

